# Listening efficiency in adult cochlear-implant users compared with normally-hearing controls at ecologically relevant signal-to-noise ratios

**DOI:** 10.3389/fnhum.2023.1214485

**Published:** 2023-07-14

**Authors:** Francisca Perea Pérez, Douglas E. H. Hartley, Pádraig T. Kitterick, Adriana A. Zekveld, Graham Naylor, Ian M. Wiggins

**Affiliations:** ^1^National Institute for Health and Care Research (NIHR) Nottingham Biomedical Research Centre, Nottingham, United Kingdom; ^2^Hearing Sciences, Mental Health and Clinical Neurosciences, School of Medicine, University of Nottingham, Nottingham, United Kingdom; ^3^Nottingham University Hospitals NHS Trust, Nottingham, United Kingdom; ^4^National Acoustic Laboratories, Sydney, NSW, Australia; ^5^Amsterdam UMC, Vrije Universiteit Amsterdam, Otolaryngology Head and Neck Surgery, Ear and Hearing, Amsterdam Public Health Research Institute, Amsterdam, Netherlands

**Keywords:** listening efficiency, listening effort, speech intelligibility, cochlear implants, ecological relevance, linear ballistic accumulator, evidence accumulation model, decision-making model

## Abstract

**Introduction:**

Due to having to work with an impoverished auditory signal, cochlear-implant (CI) users may experience reduced speech intelligibility and/or increased listening effort in real-world listening situations, compared to their normally-hearing (NH) peers. These two challenges to perception may be usefully integrated in a measure of listening efficiency: conceptually, the amount of accuracy achieved for a certain amount of effort expended.

**Methods:**

We describe a novel approach to quantifying listening efficiency based on the rate of evidence accumulation toward a correct response in a linear ballistic accumulator (LBA) model of choice decision-making. Estimation of this objective measure within a hierarchical Bayesian framework confers further benefits, including full quantification of uncertainty in parameter estimates. We applied this approach to examine the speech-in-noise performance of a group of 24 CI users (M age: 60.3, range: 20–84 years) and a group of 25 approximately age-matched NH controls (M age: 55.8, range: 20–79 years). In a laboratory experiment, participants listened to reverberant target sentences in cafeteria noise at ecologically relevant signal-to-noise ratios (SNRs) of +20, +10, and +4 dB SNR. Individual differences in cognition and self-reported listening experiences were also characterised by means of cognitive tests and hearing questionnaires.

**Results:**

At the group level, the CI group showed much lower listening efficiency than the NH group, even in favourable acoustic conditions. At the individual level, within the CI group (but not the NH group), higher listening efficiency was associated with better cognition (i.e., working-memory and linguistic-closure) and with more positive self-reported listening experiences, both in the laboratory and in daily life.

**Discussion:**

We argue that listening efficiency, measured using the approach described here, is: (i) conceptually well-motivated, in that it is theoretically impervious to differences in how individuals approach the speed-accuracy trade-off that is inherent to all perceptual decision making; and (ii) of practical utility, in that it is sensitive to differences in task demand, and to differences between groups, even when speech intelligibility remains at or near ceiling level. Further research is needed to explore the sensitivity and practical utility of this metric across diverse listening situations.

## 1. Introduction

Cochlear-implants can partially restore hearing function to people with severe-to-profound hearing loss (HL). Their effectiveness has been widely proven in terms of improving outcomes such as speech recognition, overall quality of life (QOL), long-term wellbeing, and mental health (Buchman et al., 2020). However, not all CI recipients achieve the same level of performance in terms of speech intelligibility (Boisvert et al., 2020). Such variability is usually attributed to individual factors such as the duration of HL, age of implantation, and the duration of CI use, among others. These factors, however, can only explain a small proportion of such variability (Zhao et al., 2020; Goudey et al., 2021). Regardless of any individual differences, the limitations of the CI technology impose additional challenges to speech perception, especially in noisy environments. The impoverishment of the auditory signal provided by implants (i.e., reduced spectral resolution, spectral smearing, absence of temporal fine structure cues, acoustic dynamic range compression) can hinder the ability to distinguish and segregate sounds, making listening a highly taxing task (Fu and Nogaki, 2005; Başkent, 2012; Winn et al., 2015). Moreover, these limitations can be exacerbated by individual factors including age, and other biological constraints (auditory nerve degradation and cochlear dead regions). Indeed, the selective attention needed to stay focused on a desired speech target while ignoring irrelevant competing sounds, could lead CI users to experience elevated listening effort (Wild et al., 2012; Strauss and Francis, 2017). Certainly, previous research has found that CI users report experiencing high levels of listening effort and fatigue in everyday life (Alhanbali et al., 2017; Hughes et al., 2018; Rapport et al., 2020). This ongoing demand for increased mental exertion could have negative consequences for communication (Hétu et al., 1988), social participation (Kramer et al., 2006; Nachtegaal et al., 2009; Pronk et al., 2011; Mick et al., 2014; Shukla et al., 2020), and long-term cognitive health (Lin et al., 2013; Pichora-Fuller et al., 2015).

In recent years, the assessment and understanding of listening effort has become a priority for the hearing science community (McGarrigle et al., 2014; Pichora-Fuller et al., 2016; Francis and Love, 2019). Different measures have been proposed to estimate the amount of effort exerted in a listening task. These measures are usually classified as physiological (brain activity and measures of the autonomic nervous system), subjective (self-reported and subjective assessments), cognitive (working memory and attention allocation) and behavioral (dual-task performance). Although these measures are sensitive to changes in participants cognitive load and could index the construct of listening effort, nowadays there is no standard method of measuring it (Rudner et al., 2012; McGarrigle et al., 2014; Alhanbali et al., 2019; Francis and Love, 2019). This is mainly because these measures are believed to assess different underlying domains of the listening effort phenomenon (Strand et al., 2018; Alhanbali et al., 2019; Francis and Love, 2019; Lau et al., 2019).

Behavioral measures are perhaps the objective assessments most commonly used to evaluate listening effort due to their simplicity and feasibility, i.e., the tasks are easy to design, implement, and perform, and no special equipment is required. These assessments are based on measurements of accuracy in task performance and speed of processing, often in the context of single or dual-task paradigms (Larsby et al., 2005; McGarrigle et al., 2014). Most commonly, response time (RT) is measured as the rate at which a cognitive task can be performed with reasonable accuracy (Phillips, 2016). Behavioral assessments assume that both accuracy and speed of processing are reduced as the level of task difficulty increases. Previous research has considered (dual-task) behavioral measures to be effective assessments of listening effort (Gatehouse and Gordon, 1990; Deary, 1994; Hällgren et al., 2001; Larsby et al., 2005; Houben et al., 2013). Their use has even been proposed in clinical settings (Gosselin and Gagné, 2010; Kaplan Neeman et al., 2022). Nonetheless, as with other listening effort assessments, measures of accuracy and RT need to be taken into account in combination since it is known that listening effort can still be experienced even when intelligibility performance is at or near ceiling (Houben et al., 2013; Pals et al., 2020; Winn and Teece, 2022).

Considerable effort has been made in the field of experimental cognitive psychology to integrate both behavioral measures, accuracy and RT, into a combined metric (Hughes et al., 2014; Vandierendonck, 2017; Liesefeld and Janczyk, 2019). In hearing sciences, this metric is usually interpreted as “listening efficiency” and considers both performance and response time in a listening task (Prodi et al., 2010; Prodi and Visentin, 2015; Visentin et al., 2017). Such integration is sought/preferred due to its consistency in terms of test-retest reliability which is greater than the ordinary analysis of response time and accuracy separately, which ignores any speed-accuracy trade-off (SATO) (Salthouse and Madden, 2008; Vandierendonck, 2017; Bakun Emesh et al., 2021). Some of the linear transformations proposed to combine both measures include metrics such as: the inverse efficiency scores (Townsend and Ashby, 1983), the rate correct score (Woltz and Was, 2006), the Linear Integrated Speed–Accuracy Score (Vandierendonck, 2017), the bin score (Hughes et al., 2014), and the listening efficiency (Prodi et al., 2010; Prodi and Visentin, 2015; Visentin et al., 2017). However, these linear transformations do not consider the curvilinear relationship between speed and accuracy. Therefore, the estimations that they provide, although accurate in some cases, have some limitations that could lead to biased or noisy results when assessing both individual differences and group comparison (Stafford et al., 2020; Bakun Emesh et al., 2021).

To overcome this limitation, in this article a RT decision model is proposed to perform a joint analysis of behavioral measures. Models of decision-making are able to characterize the SATO that is inherent to decision-making processes (Forstmann et al., 2011; Heitz, 2014; Stafford et al., 2020). Such models not only provide a combined analysis of speed and accuracy data but also offer increased statistical power (Stafford et al., 2020). Their use has become predominant in the cognitive psychology field and their effectiveness in characterizing the underlying processes of rapid decision tasks has been widely demonstrated (Gomez et al., 2007; Forstmann et al., 2010, 2011, 2016; Heathcote and Love, 2012; Evans and Wagenmakers, 2020).

**FIGURE 1:**
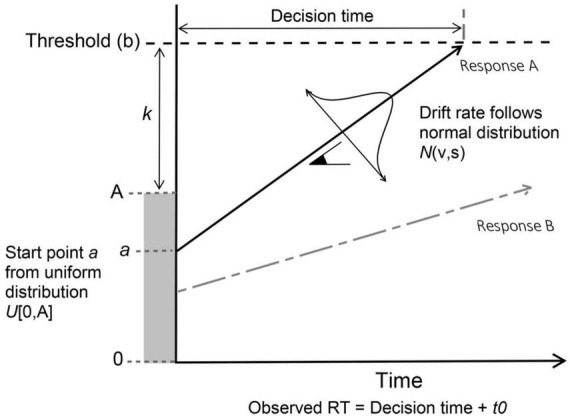
Graphical representation of the accumulation process assumed by LBA model, created based on Donkin et al. (2011) and Nishiguchi et al. (2019) studies. Racing LBA accumulators representing hypothetical responses A and B, where A is the selected response that first reached the evidence threshold (b).

The main assumption behind these models is that decisions are made when enough evidence is accumulated in favor of a particular response option. Among all accumulator models available, we use here a hierarchical linear ballistic accumulator model (LBA: Brown and Heathcote, 2008). The LBA is a simplified version of these cognitive models and is classified as a race sequential sampling model in which the accumulation of evidence occurs linearly over time toward a common response threshold. They can be used to predict both response probabilities and response times in speeded decision-making paradigms. All possible choices (either binary or multiple) are represented with independent evidence accumulators that gather evidence for each response. In this way, the decision made corresponds to the accumulator that first reaches the response threshold (Figure 1). The observed RT is assumed to be the sum of the decision and non-decision time. The decision time is the amount of time taken for the faster accumulator to reach the threshold, while the non-decision time (t0) is a constant value representing other non-decision processes.

The LBA model comprises different parameters that are related to different components of the underlying cognitive process that occur during the decision making (Evans and Wagenmakers, 2020). These parameters shown in Figure 1 are: (1) the drift rate (v) which is the speed of evidence accumulation for each response option and is able to reflect both task difficulty and participants’ efficiency in information processing; (2) the decision threshold (b) is the amount of evidence required to trigger a decision and is able to reflect task caution; (3) the starting point (a) is the amount of evidence that already exist for a particular response even before the accumulation of evidence starts, and represents any response bias that participants may have toward a particular response; (4) the non-decision time (t0) is the amount of time needed for other processes not related to the decision making, such as the speed of perceptual encoding (of a given stimulus) and response execution (e.g., button pressing); (5) the response caution, calculated as K + A/2, is the amount of evidence required to reach a decision (response).

The LBA approach therefore allows dissociating RTs into the different processes that are involved in a response decision. Moreover, it uses hierarchical Bayesian statistics which provides clear advantages in estimating parameters at both individual and group levels (Robert, 2007b; Nilsson et al., 2011; Katahira, 2016; Liu et al., 2017). Individual parameter estimates are constrained by group-level distributions, assuming that participants within each group are similar, but not identical to each other. Therefore, the model accounts for individual differences while identifying group commonalities. The probabilistic nature of the Bayesian approach also offers the ability to quantify the uncertainty of the parameters’ estimation (Robert, 2007a; Annis and Palmeri, 2018). It considers the entire response time distribution instead of single point estimates (e.g., mean, median). Likewise, results of model’s parameters are provided as full posterior probability distributions whose credible interval is computed by the 95% Highest Density Interval (HDI), which is the shortest interval that contains 95% of the mass posterior distribution (Hyndman, 1996). In contrast to the orthodox confidence interval, one can be 95% confident that the true value of a particular parameter lies within the HDI interval (Lee and Wagenmakers, 2014). Additionally, individual-level posterior distributions can be extracted to compute correlations between model parameters and other measures of interest. Following the plausible values approach, it is possible to obtain the sample plausible correlations that can then be generalized to the wider population (plausible population correlations) (Ly et al., 2017, 2018).

To take all the advantages of this approach, this study aimed to apply a LBA model to perform the analysis of the behavioral data collected in a laboratory experiment. The experiment was designed to assess the listening effort experienced by a group of adult CI users and a group of age-matched NH controls using a wide range of methods, including self-reported, cognitive, behavioral, and physiological measures. Individual differences in cognition and everyday listening experiences were characterized by means of non-auditory working-memory and linguistic-closure tests, as well as standardized hearing questionnaires. In the main laboratory task, simultaneous behavioral, subjective, pupillometry, and brain-imaging measures were collected to assess the listening effort in an ecologically-relevant speech in noise task at three levels of difficulty (or signal-to-noise ratios).

It is important to note that the objective of the study is not to demonstrate the advantages of decision models over the analysis of RT and accuracy separately given that previous research has already addressed this (Donkin et al., 2009; White et al., 2016; Stafford et al., 2020; Bakun Emesh et al., 2021). Instead, we exploit LBA analysis to obtain a single metric that objectively characterizes participants’ performance during listening tasks. We took as our primary performance metric the net drift rate since it provides an integrated estimation of the relationship between response time and accuracy (Donkin et al., 2009), and thus reflects both intelligibility and listening effort. We proposed this metric as a putative marker of participants’ listening efficiency and hypothesized that it would be sensitive to changes in listening performance across groups (between-subjects) and conditions (within-subjects). We expected that CI users would show inferior listening efficiency and greater self-reported effort than NH controls in the laboratory speech-in-noise task, as well as report less positive listening experiences than controls in questionnaires assessing daily life. Moreover, correlations between listening efficiency and participants’ cognitive and subjective scores were explored to determine whether these could act as individual predictors of listening efficiency.

## 2. Materials and methods

The study was approved by the University of Nottingham Research Ethics Committee (reference: 247-1902).

### 2.1. Participant recruitment

Recruitment was carried out primarily through the Nottingham Biomedical Research Centre (Hearing Theme) Participant Database. The study was also advertised by national and regional hearing charities and organizations in the United Kingdom including the Royal National Institute for Deaf People^1^ and the National Cochlear Implant Users Association.^2^

A group of 24 CI recipients and a group of 25 age-matched NH controls volunteered to take part in the study (participant demographics in Section “3.1. Participant demographics and hearing profile”). All participants were adults (aged 18 or over), right-handed as assessed using the Edinburgh Handedness Inventory (Oldfield, 1971), English native speakers, with normal or corrected-to-normal vision (e.g., glasses), and no history of motor (e.g., cerebral palsy) or cognitive impairment (e.g., dementia or brain injury). Participants in the CI group were required to have at least 6 months of experience using their implant(s), and were tested in their best aided condition (e.g., with a HA in the contralateral ear if bimodal listeners). Participants in the NH group were confirmed to have normal hearing with a pure-tone audiometry air-conduction hearing screen (≤20 dB HL pure-tone average across 0.5, 1, 2, and 4 kHz). This criterion was relaxed to 30 dB HL PTA for participants over 60 years of age, but only three participants exhibited a PTA worse than 20 dB HL. After providing informed consent, participants completed the experiment that lasted approximately 2 h. Participants received an inconvenience allowance of £7.50 per hour and local travel expenses were covered (up to a maximum of £15).

### 2.2. Test procedure

After pure tone audiometry (PTA) and a short interview about participants’ implantation experience (patient group only) were conducted, participants completed digitized versions of hearing questionnaires (SSQ12, EAS, FAS and HHQ) at their own pace on a touchscreen device. These questionnaires were administered to enquire about participants’ daily life experiences. Additionally, participants were asked to perform Reading Span (RSpan) and Text Reception Threshold (TRT) tests to characterize their working memory and linguistic abilities.

**FIGURE 2:**
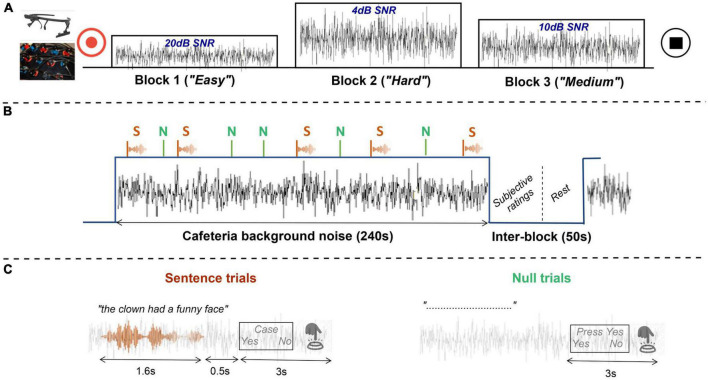
Schematic representation of the speech-in-noise task. **(A)** Example of an experimental run, whose blocks or experimental conditions are presented in easy, hard, and medium order (after randomization). Physiological measures (fNIRS and pupillometry) are recorded for the duration of the entire run. **(B)** Example of an experimental block, where sentence (*S*) and null trials (*N*) randomly presented, are masked by a continuous cafeteria background noise. During the inter-block pause, participants submit their subjective ratings. **(C)** Example of sentence and null trials with their corresponding tasks: indicating whether a probe word was featured in the sentence or submitting a specific response as instructed in null trials. Both trial types have approximately the same duration.

The main laboratory task was a hybrid block-event design divided into two runs where simultaneous behavioral, pupillometric, and optical brain-imaging measures were collected. Each run lasted approximately 14 min and consisted of three blocks of 4 min representing the three auditory experimental conditions (Figure 2). These listening conditions established the three levels of difficulty considered in the experiment and were defined by the SNR as; Easy (20 dB), Medium (10 dB), and Hard (4 dB). Each block contained 28 trials. The trials’ stimulus-onset was randomly varied in the range of 6–10 s. Moreover, no trial occurred within the first 10 s of a block. In this way, participants were given some time to acclimatize to the background noise before the presentation of any trial.

There were two types of trials, sentence and null trials, each appearing 18 and 10 times per block, respectively, in random interleaved order. Each trial was comprised of a single sentence roughly 1.6 s in length, a post stimulus pause of 0.5 s and a yes/no decision task (Figure 2). In sentence trials, participants were instructed to listen to speech sentences masked by a continuous background noise. Then, they had to answer by pressing a button a simple yes/no question, whether a probe word (presented visually on screen) was featured in the sentence just heard. Participants were encouraged to answer as accurately and quickly as possible and make their best guess when they were in doubt. Participants had 3 s to indicate their answer; otherwise, a missed response was recorded. The probability that the probe word had featured in the sentence was 50%, while in the remaining 50% of the trials a foil word was presented. Foil words were chosen to rhyme with the keyword, and, where possible, to be semantically plausible (e.g., in the sentence “The green tomatoes are small,” the keyword “green” might have been replaced with the foil word “clean”). In null trials, the sentence was muted and only the noise was audible. In those cases, participants were instructed to submit a specific response either “press yes” or “press no.” Null trials acted as a noise baseline needed for the interpretation of brain imaging measures.

A silent baseline of approximately 50 s was also included between blocks. During this time and immediately after each block, participants were asked to report on their subjective listening experience during that block using visual analog scales. Participants could respond anywhere along the 10 cm scale, with no intermediate marks or labels. The three questions that provided participants’ task subjective scores were:

– Q1. Perceived effort: “How much effort was needed to understand the sentences?” (endpoints: “No effort” and “Extreme effort”).
– Q2. Perceived intelligibility: “How many of the sentences did you understand?” (endpoints: “None of the sentences” and “All of the sentences”).
– Q3. Task disengagement: “How often did you give up trying to understand the sentences?” (endpoints: “Never gave up” and “Always gave up”).

Participants had 40 s to give their answers using a mouse as the input device. After this period, the questions disappeared, recording missed answers if no rating was reported. The total duration of the main task was approximately 30 min, however, a break between runs was always offered for participant comfort. During testing, the researcher observed and took notes from a control room adjacent to the sound booth where the main task was performed.

A short practice session was conducted before commencing data collection in which participants gained familiarity with the task and stimuli. During this practice, the researcher was present in the testing room to provide additional support when needed. The practice was designed to gradually instruct participants to perform the task. The practice session was conducted before the fNIRS and eye-tracker equipment were placed on the participant’s head. All experimental programming was implemented in Matlab (MATLAB R2018b, The MathWorks Inc., Natick, MA, USA).

A momentary fatigue questionnaire (MFQ) was also completed by participants before and immediately after the main task to assess any change in participants’ state of fatigue as a result of performing the task. Participants answered the question “How much fatigue (tiredness, weariness, problems thinking clearly) do you feel right now?” by putting a cross by hand anywhere on a numeric visual analog scale divided in equal sections from 0 to 10, 0 being labeled as “None at all” and 10 as “Extreme fatigue.”

### 2.3. Materials and stimuli

The following tests and questionnaires were considered appropriate within the context of the experiment to characterize participants’ cognitive abilities and listening experiences. These assessments were chosen due to their practicality; they are concise, intuitive, and easy to administer.

#### 2.3.1. Hearing questionnaires

• The effort assessment scale (EAS) measures self-reported listening effort in daily life of people with HL (Alhanbali et al., 2017). It consists of six questions whose responses are provided on a visual analog scale from 0 indicating “No effort” to 10 “Lots of effort.” Participants put a mark at any point of the scale that best represent their experiences. The ratings from all questions are adding up to obtain the final EAS score, which is expressed in a range between 0 and 60, with higher scores indicating more effort.
• The fatigue assessment scale (FAS) (Michielsen et al., 2004) aims to measure fatigue in a general domain. It is formed of 10 short statements that are rated on a five-point likert scale divided into five answer categories: 1 = Never, 2 = Sometimes; 3 = Regularly; 4 = Often and 5 = Always. The scale score is calculated by summing up all items, having into account that items 4 and 10 require reverse scoring. The total score of FAS ranges from 0 to 40, with higher scores indicating more fatigue.
• The short version of the Speech, Spatial and Qualities of Hearing Scale (SSQ12) (Noble et al., 2013) measures hearing ability and consists of 12 questions with answers provided in a numeric visual analog scale divided in equal sections from 0 to 10. Participants give their answers, by putting a mark at any point of the scale, considering that 0 means being unable and 10 being perfectly able to do or experience what is described in the question. A “non-applicable” box was also included for participants to indicate when a particular question is not relevant to their everyday experiences. The final score is calculated by averaging all the ratings reported and it ranges from 0 to 10, with higher scores indicating better hearing abilities.
• The Hearing Handicap Questionnaire (HHQ) evaluates, in 12-items, the social restrictions and emotional distress caused by hearing impairment. Responses are scored using a five-point scale with equal intervals (almost, always, often, sometimes, rarely, never). All responses are averaged and scaled to provide a final handicap score that ranges from 0 to 100, with higher scores indicating greater handicap.

#### 2.3.2. Cognitive tests

Two cognitive tests were used to assess individual differences in cognitive and linguistic abilities. Both tests were selected as they involve a non-auditory task that allows comparisons across participants and groups, regardless of their hearing status.

• The Reading Span Test (RSpan test) measures verbal working memory capacity with written stimuli (Daneman and Carpenter, 1980). Baddeley et al. (1985)’s version was used in the study and consisted of the presentation of five-word sentences with a subject-verb-object syntax (e.g., “The captain” “sailed” “his boat”), half of which were semantically incorrect (e.g., “The train” “sang” “a song”). Participants were asked to read each sentence aloud and judge the semantic correctness of each sentence immediately after presentation. The sentences were grouped in three blocks of three, four, and five sentences, respectively. After each block of sentences was presented, participants were asked to recall in the correct order either the first or the last words of every sentence in that block. A total of thirty-six sentences were presented in nine blocks. Prior to the test, one block of three sentences was presented in a practice session for participants to become familiar with the task. The Rspan score is the proportion of words that participants were able to recall correctly, and thus higher scores indicate greater working memory capacity.
• The Text Reception Threshold (TRT) test measures the “linguistic closure” ability to integrate and complete partially masked sentences (Zekveld et al., 2007). The test consists of reading aloud digitally presented sentences that are partly masked by a bar pattern. The text that is not covered by the bar pattern represents the percentage of unmasked text that is modified throughout the test in an adaptive procedure, increasing or decreasing in 6% steps, according to participants’ responses. No feedback was given during the test and participants were encouraged to make their best guess when in doubt. The TRT score is defined by the average percentage of unmasked text required to read 50% of the sentences correctly. Lower TRT scores indicates better performance.

Participants completed one practice session with 10 sentences and two TRT tests with 16 sentences each. For consistency, the sentences were presented in the same order to all participants and were obtained from three Bamford-Kowal-Bench (BKB) sentence lists, which were only used for the TRT test. Note that this experiment used a slightly modified version of the test, which was adapted and provided by Zekveld et al. (2007). This version only scores key words within each sentence (three key words per sentence), in this way importance is given to words that provide more meaning within the context. For instance, if a non-key word (e.g., “the”) was not read correctly but all key words of the sentence were, then the overall sentence was scored as “correct.” The final rating was calculated for each participant as the averaged TRT score between the two tests performed.

#### 2.3.3. Speech material and background noise

A real-world recording of a busy atrium café from the RealSpeech content library was used (with permission of Dr. Ian Wiggins and Dr. Mark Fletcher) as background noise. This “cafeteria” background noise was used to mask the target speech sentences. The difficulty of the listening task was manipulated by varying the level of speech relative to the level of the background noise (SNR), defining the three experimental conditions:

– Easy: +20 dB SNR (Speech level 65 dBA, noise level 45 dBA).
– Medium: +10 dB SNR (Speech level 65 dBA, noise level 55 dBA).
– Hard: +4 dB SNR (Speech level 65 dBA, noise level 61 dBA).

Each condition was presented in a separate block (i.e., the SNR was kept fixed throughout each 4-min block). The conditions were randomly presented for each run and participant. To avoid startling participants, the background noise was faded in and out gradually at the start and end of each block (fade duration 3 s).

The type of background noise and SNRs assigned for each condition were chosen to be representative of everyday life sound scenarios. Particularly, the SNRs were selected based on previous studies that characterized the most commonly found real world SNRs of older adults with mild-to-moderate hearing loss (Smeds et al., 2015; Wu et al., 2018). For instance, +4 dB SNR (the hard condition), was the average most common SNR found for “noisy” speech listening situations. Likewise, +10 dB SNR (the medium condition), was described to be the median SNR found in different listening environments, such as “home,” “indoors other than home,” and “outdoors.” Finally, +20 dB SNR (the easy condition) was the most favorable condition found in everyday life scenarios and characterized as “very quiet situations.” Unlike previous studies, the SNRs used in the main task were not adjusted to each participant. This was intended to provide greater “realism” to the experiment, since people are not usually able to modify the noise levels encountered in real life scenarios.

Speech material consisted of recordings of BKB sentences (Bench et al., 1979) spoken by a male talker. Seventeen sentence lists were available, each comprising 16 sentences. For each participant, a random subset of the available lists was selected and the sentences from those lists were randomly assigned to the three experimental conditions for each run. No participant was presented with the same BKB sentence more than once during the entire testing session. Prior to use, the sentences were convolved with the impulse response of the space in which the background noise was recorded. By doing so, the acoustic characteristics of the space, in particular the reverberation time (∼1.4 s), were applied to the target speech sentences.

### 2.4. Equipment

A touchscreen laptop connected to an external monitor was used to conduct the hearing questionnaires and cognitive tests. For this part of the experiment, participants were seated in the control room at approximately 45 cm from the external monitor, where the sentences were displayed.

The main behavioral task was conducted in a sound-attenuated room. Participants were seated at approximately 75 cm from a display screen with a loudspeaker (Model 8030A, Genelec, Iisalmi, Finland) mounted immediately above it. Auditory stimuli were presented in the free field. The sound pressure levels were measured at the listening position using a Brüel & Kjaer Type 2250 sound level meter. Participants entered their responses using a mouse and a “RTbox” button box (Li et al., 2010).

### 2.5. Statistical analysis

Participants’ subjective scores during the task (perceived effort, intelligibility, and task disengagement) were calculated per each listening condition and averaged across runs using Matlab (MATLAB R2018b, The MathWorks Inc., Natick, MA, USA). Likewise, cognitive tests scores (RSpan and TRT tests) were calculated by custom applications implemented in Matlab and Delphi, respectively. These scores together with those resulting from the hearing and momentary fatigue questionnaires were analyzed in RStudio (Version 4.1.2; R Core Team, 2021). To facilitate comparison of hearing and cognitive test scores, reverse scoring was applied to the SSQ12 questionnaire and RSpan test so that greater scores represent worse hearing ability and less working memory capacity, respectively.

Group level differences were examined by computing Bayesian analyses using the R package brms (Bürkner, 2017). The brms package implements Bayesian multilevel models using the probabilistic programming language Stan. The formula used to analyze hearing questionnaires, cognitive tests, momentary fatigue, and group age differences, regressed each outcome variable on the effect of Group (e.g., Age ∼ Group), assuming unequal variances of both groups (e.g., sigma ∼ Group). On the other hand, the formula for task subjective measures assessed the interaction “group per condition,” taking into account participants’ random effects by group {EF ∼ 0 + Intercept + Group:Condition + [1 | gr(Participant, by = Group)]}. Ordered beta regression (Kubinec, 2022) was set as the custom family distribution to model participants’ responses to hearing questionnaire, cognitive tests and task subjective measures. Such distribution was explicitly designed for survey data where slider and visual analog scales (with both lower and upper bounds) are used. The Gaussian family was chosen to perform linear regression for outcome variables such as age and momentary fatigue measures. The latter was analyzed on the difference post-vs. pre experiment momentary fatigue scores. Prior distributions for ordered beta regression models were set as defined by Kubinec (2022), whereas default flat priors were used on the effect of age and momentary fatigue scores. Posterior distributions were estimated using the Markov Chain Monte Carlo (MCMC) (van Ravenzwaaij et al., 2018) algorithms, whose convergence was measured by the potential scale reduction factor R-hat(R) (Brooks and Gelman, 1998) over four separate chains, each with 2,000 warmup iterations followed by another 2,000 post-warmup iterations. Posterior predictive checks were performed to ensure that the models’ predictions adequately fit the data.

The model conditional effects, predicted means and 95% credible intervals (CrI), were reported per group and condition (when relevant). Effects sizes were also calculated, when relevant, using Cliff’s Delta statistics on the posterior distribution of the model’s predicted data. This non-parametric effect size measure was chosen for its suitability to analyze ordinal data (Likert scales), which reduces the influence of outliers or groups’ variance differences.

A hierarchical Linear Ballistic Accumulator model (LBA) was performed to analyze participants’ behavioral responses following Gunawan et al. (2020). This approach allows modeling intercorrelated individual level LBA parameters using a multivariate normal prior distribution. The LBA model was implemented in Stan as described by Annis et al. (2017), using a non-centered parameterization to efficiently explore the posterior parameters’ distributions. Missing response times (late responses >3 s) were treated as parameters estimated within the model, assuming that their response accuracy would have been at chance level. The model scaling constraint was set so that the between-trial variability in the drift rates was fixed to one (sv = 1). The model considered fifteen free parameters, two evidence accumulators, one per each response option (correct vs. incorrect), that were allowed to vary across SNR conditions (Easy, Medium, Hard) and trial type (Sentence vs. Null). The remaining parameters, A, b and t0 were allowed to vary across groups but were fixed across trials. Posterior distributions were estimated using MCMC algorithm, over four separate chains, each with 1,000 warmup iterations followed by another 2,000 sample iterations (8,000 draws in total). Posterior predictive checks were used to assess the agreement between model predictions and observed data. Effects were assessed using 95% Highest posterior density intervals (HDI; HDInterval package) that acted as the 95% CrI of the average posterior parameters.

Differential drift rates were calculated per each group as the difference between correct and incorrect responses. This parameter indicates the rate at which a listener preferentially accumulates evidence toward a correct response and was considered a putative indicator of participants’ listening efficiency. Thus, faster accumulation of evidence toward correct answers was interpreted as greater listening efficiency, that is, greater ability for correctly recognizing speech. Differential drift rates were calculated for both trial types to examine whether group differences occur during the listening process (sentence trials) or task execution (null trials).

Moreover, individual-level differential drift rates in sentence trials were extracted (per participant) and averaged across conditions. A correlation analysis following the plausible values approach (Ly et al., 2017) explored relationships between participants’ listening efficiency and task subjective, cognitive, and hearing questionnaires scores. Correlations between 8,000 posterior draws per individual’s drift rates and their scores on these measures were computed, resulting in a distribution of plausible correlations. The posterior distribution of the population correlation was also calculated (Ly et al., 2018), and each group’s correlation mean and 95% CrI were reported. Correlations were considered reliable when the 95% CrI did not contain zero or when a high degree of certainty (>90%) suggested that the true population correlation (plausible p) was different from zero.

## 3. Results

### 3.1. Participant demographics and hearing profile

Both groups of participants had a similar age range; CI users: 20–84 years and NH controls: 20–79 years. The age similarity of both groups was confirmed by the results of the analysis that showed similar predicted means and overlapping 95% CrIs (CI: 60.5 [53.8, 67.1], NH: 55.9 [50, 62]) between groups. In terms of gender, both groups were similarly distributed with 10 and 11 males in the CI and the NH group, respectively.

Participants in the control group had normal (or near-normal) hearing as assessed using air-conduction PTA screen across frequencies 0.5, 1, 2, and 4 kHz in both ears (22 adults with an average threshold ≤20 dB HL and three adults with an average threshold ≤30 dB HL). Participants in the CI group had an average of 8 years of CI experience (range 1–24 years). According to hearing device configuration, there were 11 bimodal listeners, 11 unilateral and 2 bilateral CI recipients. Most participants had severe-to-profound HL in the non-implanted ear as revealed by mean audiometric thresholds (in dB HL) (Unilateral: 93, 100, 100, 100; Bimodal: 83, 82, 85, 92). Likewise, low-frequency residual hearing in the implanted ear(s) was hardly preserved [Unilateral (M: 100, SD: 1.5); Bimodal (M: 95, SD: 12.3); Bilateral (M: 99, SD: 1.8) dB HL]. Twenty CI users reported having developed HL after language acquisition while four were pre-lingually deafened. In all cases, CI participants were able to perform all listening tests included in the study as well as maintain conversations and communicate effectively with the researcher. Demographic information for CI participants is listed in Table 1.

**TABLE 1:** Demographics of CI participants.

CI participant	Gender	Age (years)	CI manufacturer	Hearing devices (implanted side)	Etiology of deafness	Years CI Exp.
1	M	66	Cochlear Nucleus 6	Unilateral (R)	Virus or disease	5
2	M	66	Cochlear Nucleus 6	Unilateral (R)	Born deaf/not known	15
3	M	56	Cochlear Nucleus 7	Bimodal (R)	Not known	6
4	F	61	Cochlear Nucleus 7	Unilateral (L)	Nerve damage	5
5	F	61	Cochlear Nucleus 7	Bimodal (R)	Not known	6
6	M	38	AB	Bimodal (L)	Born deaf/Not Known	8
7	F	56	Cochlear Nucleus 7	Unilateral (R)	Not known	4
8	F	69	Cochlear Nucleus 7	Unilateral (L)	Not known	1
9	M	23	Cochlear Nucleus 7	Unilateral (R)	Ototoxicity	21
10	F	20	Med-EL	Bilateral CIs	Born deaf/not known	8
11	M	56	Cochlear Nucleus 6	Bimodal (R)	Born deaf/not known	4
12	M	84	Cochlear Nucleus 6	Bimodal (L)	Virus or disease	3
13	F	75	AB	Bimodal (L)	Virus or disease	13
14	M	66	Med-EL	Unilateral (R)	Virus or disease	8
15	F	64	Med-EL	Bimodal (R)	Genetics	9
16	F	78	Med-EL	Unilateral (R)	Virus or disease	8
17	F	73	Med-EL	Bilateral CIs	Born deaf/not known	23
18	F	72	Cochlear Nucleus 7	Unilateral (R)	Virus or disease	2
19	M	73	Cochlear Nucleus 6	Bimodal (L)	Genetics	4
20	F	53	Cochlear Nucleus 6	Bimodal (L)	Virus or disease	22
21	M	51	Cochlear Nucleus 6	Bimodal (L)	Ototoxicity	5
22	F	72	Cochlear Nucleus 7	Unilateral (L)	Not known	24
23	F	71	Cochlear Nucleus 7	Unilateral (R)	Not known	5
24	F	43	Cochlear Nucleus 7	Bimodal (L)	Not known	2

Gender: F for female and M for male. CI manufacturer: AB for Advanced Bionics. Implanted side: R for right and L for left. The variable “Years CI Exp.” refers to years of CI usage experience.

### 3.2. Cognitive and hearing function

**FIGURE 3:**
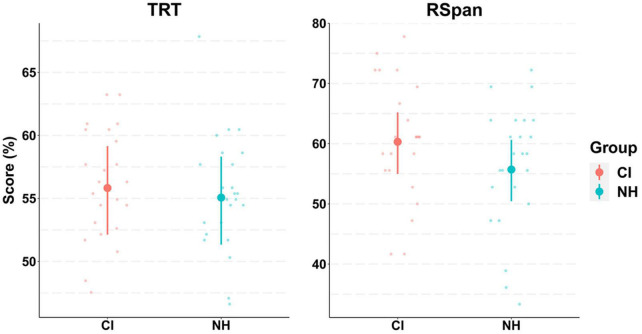
Model conditional effects over raw data for participants’ cognitive tests results by group (e.g., TRT∼ Group). Abbreviations refer to Text Reception Threshold (TRT) and Reading Span (RSpan) tests for both groups of cochlear implant (CI) and normally hearing (NH) participants. The error bars display 95% credible intervals; the bold dots represent posterior means, and the small dots represent the raw data. Scores for TRT and RSpan tests range between 0–100, with greater scores indicating worse performance. Note that RSpan scores were reversed so that greater scores represent less working memory capacity.

Participants in both groups achieved similar scores on cognitive tests (Figure 3). Although the difference in scores between groups was slightly greater in the RSpan test compared to the TRT test, the model 95% CrIs show considerable overlap between groups. The model predicted means and 95% CrIs for the RSpan test were 60.3 [54.9, 65.1] and 55.7 [50.6, 60.6], for the CI and NH groups, respectively, whereas for the TRT they were 55.9 [52.3, 59.2] and 55.1 [51.6, 58.4].

**FIGURE 4:**
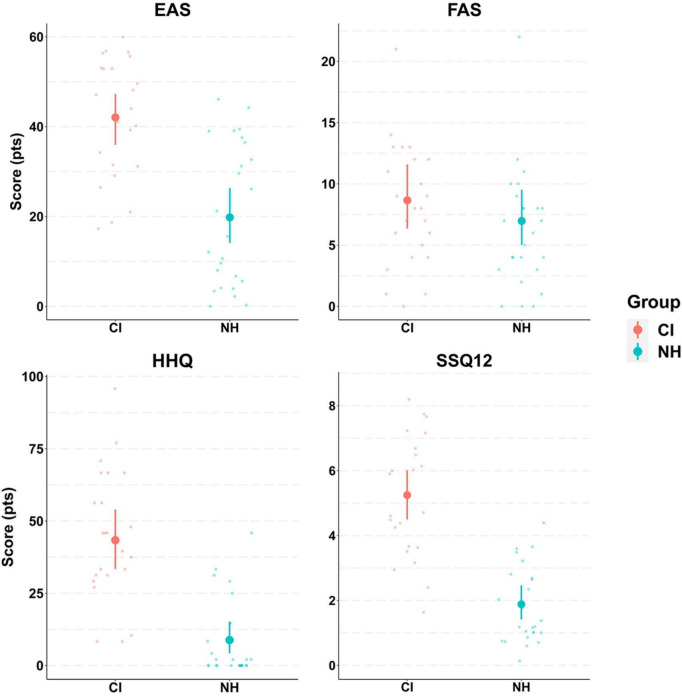
Model conditional effects over raw data for participants’ hearing questionnaires results by group (e.g., EAS∼ Group). The error bars display 95% credible intervals; the bold dots represent posterior means, and the small dots represent the raw data. The effort assessment scale (EAS) questionnaire has a score range between 0–60 points. The fatigue assessment scale (FAS) ranges between 0–40 points. The hearing handicap questionnaire (HHQ) has a score range of 0–100 points. The short version of the speech, spatial and qualities of hearing scale (SSQ12) was reverse scored, with a total range of 0–10 points. Greater scores in all questionnaires indicate greater hearing difficulty.

The results of hearing questionnaires, however, showed greater differences between groups, with CI users reporting greater hearing difficulties on all questionnaires compared to their NH peers (Figure 4). Strong effects of group were evident in the EAS, HHQ, and SSQ12 as revealed by non-overlapping 95% CrIs. Indeed, CI users’ EAS scores were double those reported by NH controls, suggesting that participants in the CI group were greatly affected by listening effort in daily life. Likewise, a difference of 3.3 points (out of 10) in the SSQ12 scores between groups suggested that CI users exhibited worse hearing abilities compared to their NH peers. HHQ scores showed a difference of 35 points (on a 100-point scale) between groups, which again is interpreted as higher self-perception of hearing disability and handicap of CI users compared to NH controls. The model predicted means and 95% CrIs for the CI group in these questionnaires were: EAS (42.1, CrI:[36, 47.3]); FAS (8.7, CrI:[6.3, 11.6]); HHQ (43.4, CrI:[33.3, 53.7]); and SSQ12 (5.2, CrI:[4.5, 6]). The NH group predicted results in these questionnaires were: EAS (19.9, CrI:[14.3, 26.1]); FAS (7, CrI:[5, 9.4]); HHQ (8.8, [4.3, 15.4]); and SSQ12 (1.9, CrI:[1.4, 2.4]).

Overall, low scores of fatigue were reported by participants before [CI (M: 2.7, SD: 1.9); NH (M: 1.5, SD: 1.8)] and after [CI (M: 4.7, SD: 2.8); NH (M: 2.8, SD: 2.2)] performing the main laboratory task. The change in participants’ state of fatigue due to the experiment was similar in both groups as revealed by the model predicted means and 95% CrIs (CI: 2 [1.2, 2.8], NH: 1.3 [0.8, 1.8]).

**FIGURE 5:**
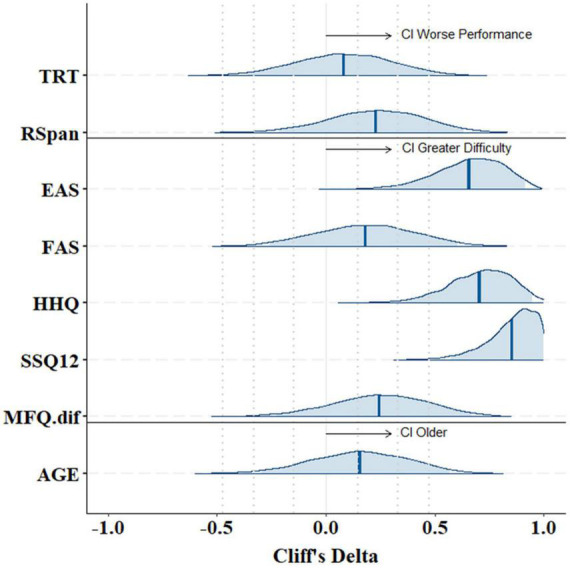
Group-difference Cliff’s Delta effect sizes with 95% credible intervals on the posterior distributions of cognitive tests (TRT, RSpan), hearing questionnaires (EAS, FAS, HHQ, SSQ12, MFQ.dif), and participants’ age. Positive Cliff’s Delta values indicate greater scores/results of participants in the CI group compared to the NH group (CI scores > NH scores). Abbreviations refer to Text Reception Threshold (TRT), Reading Span (RSpan) tests, Effort Assessment Scale (EAS), Fatigue Assessment Scale (FAS), Hearing Handicap Questionnaire (HHQ), short version of the Speech, Spatial and Qualities of hearing scale (SSQ12), and Momentary Fatigue Questionnaire (MFQ.dif) on the difference post-vs.-pre experiment.

Cliff’s Delta effect size calculation (Figure 5) confirmed large group effects in EAS (*d* = 0.7), HHQ (*d* = 0.9), and SSQ12 (*d* = 0.7) questionnaires, with group-difference posterior distributions that do not contain zero. This suggests that CI users experienced significantly greater hearing difficulty in daily life compared to NH controls. Cognitive measures, however, showed a weak effect of group on both the TRT (*d* = 0.08) and RSpan (*d* = 0.2) tests, suggesting that on average participants in both groups exhibited similar working memory capacity and linguistic closure abilities. Likewise, the effect of group on fatigue scores was small in both the FAS (*d* = 0.2) and the MFQ post-vs.-pre-experiment (*d* = 0.25). These weak effects were also associated with a large uncertainty as expressed by wider credible intervals.

### 3.3. Task subjective results

#### 3.3.1. Self-reported listening effort

**FIGURE 6:**
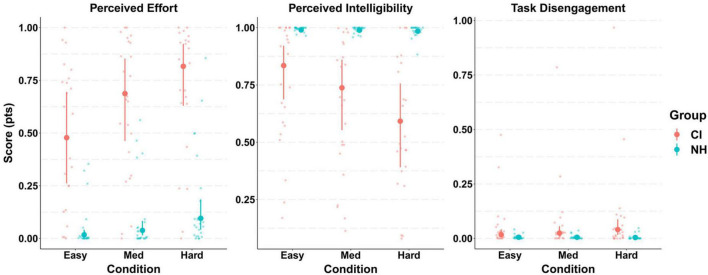
Model conditional effects over raw data for participants’ task subjective ratings (self-perceived listening effort, intelligibility, and task disengagement) by Group and Condition {e.g., EF ∼ 0 + Intercept + Group:Condition + [1 | gr(Participant, by = Group)]}. The error bars display 95% credible intervals; the bold dots represent posterior means, and the small dots represent the raw data. Scores were measured in a 0–1 scale.

Participants in the CI group reported greater levels of perceived listening effort during the behavioral task compared to those in the NH group in all SNR conditions. The model predicted means (Easy [CI M: 0.48, NH M: 0.02], Medium [CI M: 0.69, NH M: 0.04], Hard [CI M: 0.82, NH M: 0.1]) and 95% CrIs on the interaction “Group x Condition” confirmed this as shown in Figure 6. Such a strong effect of group was evident as revealed by 95% CrIs that did not overlap in any condition. These results suggest that CI users perceived significantly greater listening effort than NH controls at all task difficulty levels. Although more dramatic in the patient group, the increasing trend in participants’ perceived effort as SNR worsened was present in both groups.

#### 3.3.2. Self-reported intelligibility

The analysis of task perceived intelligibility also indicated considerable differences between both groups of participants (Figure 6). A ceiling effect was observed in the NH group in all experimental conditions as shown by the model predicted means (Easy M:0.99, Med M:0.99, Hard M:0.98), with extremely narrow 95% CrIs (± 0.01). The patient group (CI), however, reported significantly inferior levels of intelligibility in all conditions (Easy [M: 0.83, CrI: (0.69, 0.92)], Med [M:0.74, CrI: (0.655, 0.86)], Hard [M:0.59, CrI: (0.39, 0.76)]), with such difference being more dramatic as the level of task difficulty increased.

#### 3.3.3. Self-reported task disengagement

Overall, low levels of task disengagement were reported by all participants in all experimental conditions. This floor effect in task disengagement was observed in the model predicted means (Easy [CI group M: 0.017, NH group M: 0.006], Med [CI group M: 0.02, NH group M: 0.005], Hard [CI group M: 0.04, NH group M: 0.004]) and narrow 95% CrIs (Figure 6). A small effect of group was only present in the hard condition where the 95% CrIs did not overlap.

### 3.4. Behavioral results

**FIGURE 7:**
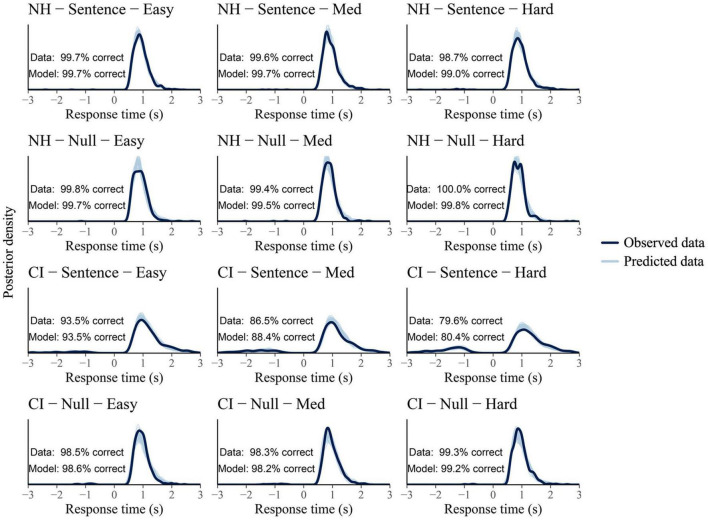
Posterior predictive checks per group (NH and CI group shown at the two **top** and **bottom** lines, respectively), trial type (sentence vs. null trials), and condition (Easy, Med, and Hard conditions shown from left to right columns). Participants’ response time (RT) for incorrect responses are plotted as negative. Solid dark lines represent the observed data and light blue lines represent the model predicted data (8,000 draws).

The LBA model was fed with 8,064 observations, corresponding to 47 participants who completed 168 trials each (two runs x three conditions x 28 trials [18 sentence + 10 null trials]), and two participants who only completed one run each (84 trials). Sixty-three observations were treated as missing responses within the model. Missing responses occurred when participants took more than 3 s to submit their answer and therefore, a not-known response was assumed. Satisfactory convergence was found for all estimated parameters as revealed by the full traces’ plots and Rhats’ range [0.99, 1.01]. Posterior predictive checks showed an adequate fit of the model’s predictive RT distributions to the observed data (Figure 7). The mean difference between predicted and observed data was 0.1% and 0.5% for the NH and the CI group, respectively, across conditions and trial types.

As can be seen in Figure 7, very few errors (plotted as negative) were made by participants during the behavioral task (on average 0.5% incorrect responses were submitted by NH participants and 9% by CI users across conditions and trial types). The high levels of accuracy were not surprising considering that the behavioral task was a yes-no task in which correctness of responses are always at least at a chance level (50%). Moreover, the listening conditions, all with positive SNRs, were considerably easy for NH listeners and more challenging for CI recipients, hence the difference in error rate between groups. To take into account this difference, the listening efficiency results are expressed using differential drift rates (vDiff = vcorrect-vincorrect).

**FIGURE 8:**
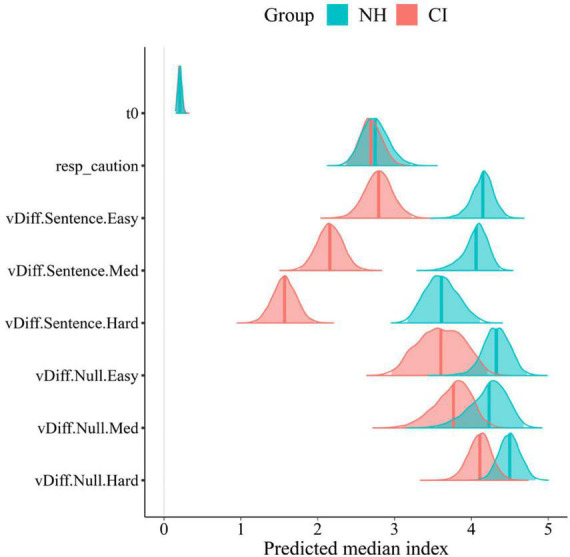
Posterior group comparison in LBA model’s parameters: t0, response caution, and differential drift rates (vDiff) per trial type (Sentence,Null), and condition (Easy, Med, and Hard). Solid lines in posterior distributions represent the predicted median index for each parameter.

Significant group differences were found in the posterior distributions of the LBA’s drift rates parameters in sentence trials (Figure 8). Overall, NH participants showed faster accumulation of evidence toward correct answers in sentence trials (greater listening efficiency) in all experimental conditions compared to CI users. Such a strong effect of group was confirmed by posterior distributions that do not overlap in any SNR condition. This can also be seen in the between-group difference column of Table 2 with means and 95% CrIs that do not contain zero. Conversely, no effect of group was found in null trials. Although CI users’ listening efficiency in null trials was slightly inferior compared to NH controls, such difference did not reach significance as revealed by the between-group difference in null trials’ drift rates (Table 2). It comes as no surprise that differences in listening efficiency between groups were only present in sentence trials, since null trial tasks did not require active listening but just following instructions instead.

**TABLE 2:** Means and Highest Density Intervals (HDI) of the LBA model’s parameters posterior distributions by group.

	NH Group			CI Group			Between-Group difference *(NH-CI)*		
	**Mean (v)**	**95% CrI** **Lower**	**95% CrI** **Upper**	**Mean (v)**	**95% CrI** **Lower**	**95% CrI** **Upper**	**Mean (v)**	**95% CrI** **Lower**	**95% CrI** **Upper**
t0	0.21	0.15	0.25	0.21	0.15	0.26	0.00	−0.08	0.07
resp_caution	2.76	2.38	3.21	2.70	2.33	3.03	0.06	−0.45	0.57
vDiff.Sentence.Easy	4.14	3.75	4.46	2.79	2.35	3.23	1.35	0.79	1.88
vDiff.Sentence.Med	4.04	3.61	4.39	2.16	1.75	2.55	1.88	1.31	2.43
vDiff.Sentence.Hard	3.62	3.18	4.12	1.57	1.20	1.95	2.05	1.45	2.65
vDiff.Null.Easy	4.32	3.86	4.74	3.60	2.97	4.22	0.72	−0.01	1.48
vDiff.Null.Med	4.20	3.59	4.70	3.74	3.13	4.26	0.46	−0.28	1.17
vDiff.Null.Hard	4.50	4.16	4.83	4.10	3.72	4.47	0.39	−0.08	0.90
vDiff.Sentence.Mean	3.93	3.67	4.20	2.17	1.89	2.48	1.76	1.35	2.14
vDiff.Null.Mean	4.34	3.99	4.67	3.81	3.42	4.18	0.52	0.05	1.02
vDiff.Sentence.Slope	−0.26	−0.54	0.02	−0.61	−0.86	−0.35	0.35	0.01	0.75
vDiff.Null.Slope	0.09	−0.08	0.29	0.25	−0.06	0.57	−0.16	−0.52	0.18

The effect of group can be seen in the between-group difference columns (last three columns). The HDI is interpreted as the 95% credible intervals (CrI) of posterior distributions. Table parameters are t0, response caution (resp_caution), and differential drift rates (vDiff). Drift rates are described in the format “vDiff. trial type. Condition” and “vDiff. trial type. Mean or Slope across conditions.”

**FIGURE 9:**
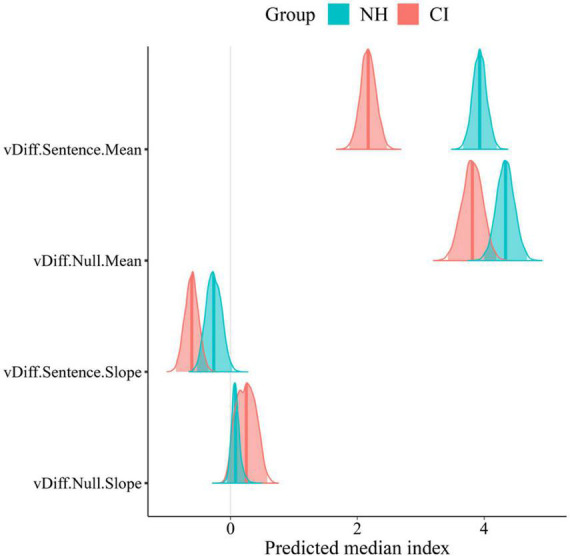
Mean value and Slope across conditions of LBA model’s differential drift rates per trial type (Sentence, Null). Solid lines in posterior distributions represent the predicted median index for each parameter.

The increase in task difficulty was also noticeable within participants’ listening efficiency across conditions in sentence trials. This effect was explored by the slope of listening efficiency across conditions (Figure 9). Negative slopes (“vDiff.Sentence.Slope” <0 in Figure 9 and Table 2) confirmed that participants’ listening efficiency in both groups was reduced as the SNR became less favorable. The effect was stronger in the CI group as revealed by steeper negative slopes across conditions (CI M: −0.61; NH M:−0.26). These results suggest that CI users may be affected to a greater extent by the worsening of SNR than their NH peers. As expected, participants’ listening efficiency in null trials was not significantly affected by the increase in task difficulty. Indeed, their performance in null trials was similar across conditions as revealed by the “zero slope” value that is contained in the 95% CrIs of both groups’ posterior distributions (“vDIff.Null.Slope” ∈ 0).

Parameters such as the non-decision time (t0) and response caution (K + A/2) were almost identical in both groups (Figure 8; Table 2). It is assumed therefore that participants in both groups had similar non-decision timings and levels of caution. In other words, they spent the same amount of time in non-decision processes and needed the same amount of evidence to make a choice.

### 3.5. Correlation analysis

**FIGURE 10:**
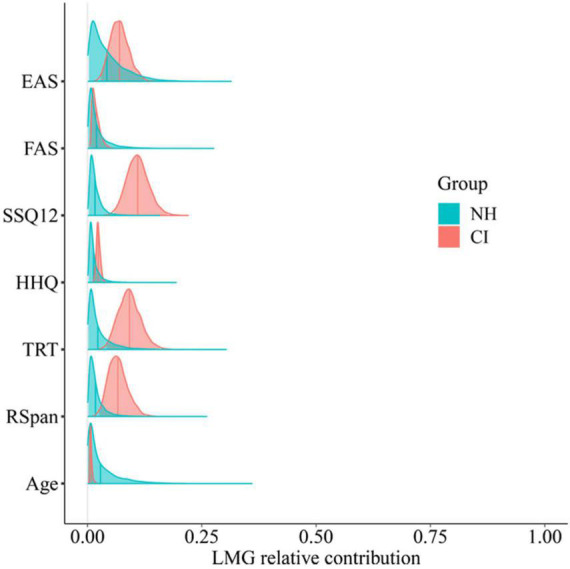
Posterior relative contribution of predictor variables to individual-sentence differential drift rate by group. Predictor variables are age, hearing questionnaires (EAS, FAS, SSQ12, and HHQ), and cognitive tests (TRT and RSpan) scores.

Relationships between participants’ listening efficiency and their scores on cognitive, subjective, and hearing questionnaires were explored following a plausible values approach. To do so, differential drift rates in sentence trials were extracted per each participant and averaged across conditions (8,000 draws each). Then, the relative importance of predictor variables (age, hearing questionnaires and cognitive scores) were calculated in a multiple linear regression model using the Relaimpo R package (Groemping, 2006). Figure 10 shows the relative contribution of these predictor variables to individual-level listening efficiency (sentence differential drift rates) by group.

As can be seen in Figure 10, none of the variables were able to explain the variance in the NH group, whereas some seemed to explain some of the variability of CI users’ listening efficiency. In particular, the SSQ12, EAS, and TRT scores accounted for 0.11, 0.07, and 0.09 of the variance, respectively. To explore any potential correlations with those variables, both plausible correlations and plausible population correlations (plausible p) for each of them were calculated following Ly et al.’s (2018) analytic approach.

**FIGURE 11:**
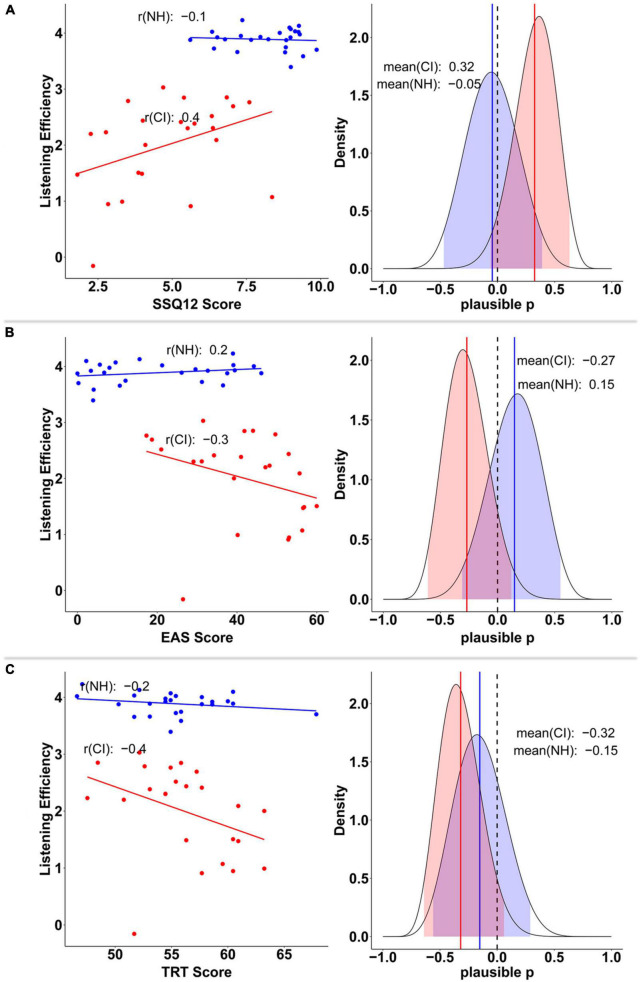
Relationship between listening efficiency and SSQ12 **(A)**, EAS **(B)**, and TRT **(C)** scores by group. Groups are plotted in red (CI) and blue (NH) colors. Plots on the left column display the posterior mean estimates of listening efficiency for each participant as a function of SSQ12, EAS, and TRT scores, respectively. Plots on the right column show the posterior distribution of the plausible population correlation (ρ) per each variable. In the later, colored solid lines and shaded areas, represent the mean, and 95% credible intervals, respectively.

Moderate-to-weak correlations were found between CI users’ scores in the SSQ12 (*r* = 0.4), EAS (*r* = −0.3), and TRT (*r* = −0.4) and their performance on the behavioral task (listening efficiency). These, although not being strong relationships, suggest that the greater the subjective hearing abilities of CI users (SSQ12 scores without reversing), the better their listening efficiency was in the behavioral task. Conversely, the more effort they reported in daily life (greater EAS scores) and the worse linguistic closure abilities they exhibited (greater TRT scores), the worse their performance was in the behavioral task (left column plots in Figure 11). Although the 95% CrIs of the plausible population correlations (plausible p) just encompassed zero (SSQ12 [−0.07, 0.64], EAS [−0.61, 0.12], and TRT [−0.65, 0.07]), there was reasonably strong evidence in favor of a correlation within the CI group. Specifically, there was 95.4% (*p* > 0), 91.7% (*p* < 0) and 95.1% (*p* < 0) certainty that participants’ listening efficiency correlated with SSQ12, EAS, and TRT, respectively (right column plots in Figure 11).

**FIGURE 12:**
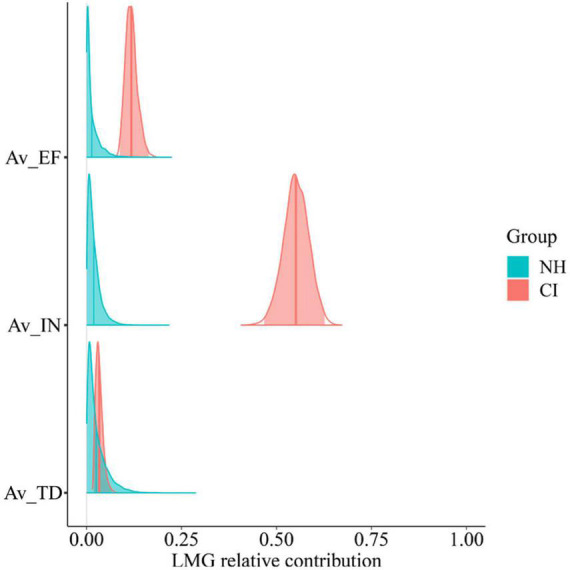
Posterior relative contribution of task subjective measures (averaged across conditions) to individual-level listening efficiency by group. Predictor variables are participants’ perceived listening effort (AV_EF), intelligibility (AV_IN), and task disengagement (AV_TD).

The relationship between participants’ subjective measures and their listening efficiency in the behavioral task was also explored. Figure 12 shows the partial contribution of these subjective measures (averaged across conditions) to the overall variability in listening efficiency.

**FIGURE 13:**
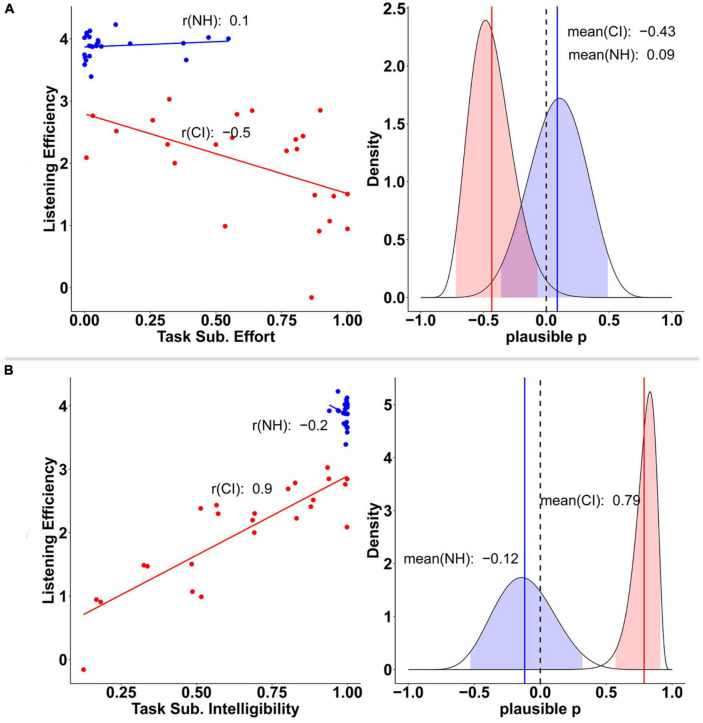
Relationship between listening efficiency and both task subjective effort **(A)**, and task subjective intelligibility **(B)** scores by group. Groups are plotted in red (CI) and blue (NH) colors. Plots on the left column display the posterior mean estimates of listening efficiency for each participant as a function of perceived effort and intelligibility, respectively. Plots on the right column show the posterior distribution of the plausible population correlation (ρ) per each variable. In the later, colored solid lines and shaded areas, represent the mean and 95% credible intervals, respectively.

Just like before, some task-related subjective measures seemed to act as individual predictors of listening efficiency only in the CI group. Both perceived intelligibility and effort contributed largely to explain CI users’ listening efficiency variability. Indeed, moderate and strong correlations were found between CI users’ listening efficiency and their subjective scores of effort (*r* = −0.5) and intelligibility (*r* = 0.9), respectively. Evidence of such correlations was also found at the population level (Figure 13) as revealed by 95% CrIs of the plausible population correlations that do not contain zero (AV_EF [−0.72, −0.06], AV_IN [0.56, 0.91]).

These results suggest that CI users’ perceived effort and intelligibility reflected accurately their listening efficiency during the task.

## 4. Discussion

In this article, a LBA model was used to perform a joint analysis of behavioral measures acquired in a laboratory experiment that aimed to assess the cognitive effort and listening performance of a group of CI users and a group of age-matched NH controls. The drift rate parameter was proposed as a putative metric of participants’ listening efficiency and its correlations with other listening effort measures (self-reported, task subjective, and cognitive) were examined following the plausible values approach.

### 4.1. CI users were disproportionately affected by moderate ecologically relevant levels of background noise

The between-group comparison revealed significant differences between CI users and NH controls as assessed by different measures of listening effort. Regarding self-reported daily life measures, EAS scores clearly showed that CI wearers reported considerably greater levels of listening effort in daily life than controls. This was consistent with their own perception of hearing abilities (SSQ12) and hearing handicap (HHQ), which again showed a clear disadvantage to CI users compared with NH participants.

Participants were also consciously aware of their effort while performing the main task. CI users reported significantly more listening effort than their NH peers did in all experimental conditions. Such remarkable difference was apparent even in the easy condition (SNR: 20dB), despite task accuracy and, self-reported intelligibility, being near ceiling in both groups (approximately 90% and 100% in CI and NH groups, respectively). These results confirm the observation already made by many researchers that listening effort can be present even at ceiling levels of speech understanding performance (Pals et al., 2013, 2020; Winn et al., 2015; Winn and Teece, 2021).

Such differences between groups were also confirmed by the behavioral results. The LBA’s drift rate parameter in sentence trials interpreted as a measure of listening efficiency showed a strong effect of group in all listening conditions.

Overall, CI users exhibited lower listening efficiency (slower accumulation of evidence toward correct answers) during sentence trials compared with NH controls in all experimental conditions. That no effect of group was found in null trials suggests that the reduction in listening efficiency observed in the CI group during sentence trials must be associated with difficulties encountered during the listening process rather than the execution of the task itself, which was the same in both trial types. This assumption is also supported by the non-decision time (t0) parameter of the LBA model and the cognitive tests’ results. Both indicating that participants had similar cognitive abilities (working memory and linguistic closure) and spent the same amount of time to perceive/encode the stimulus and execute the task (button pressing). Therefore, the increased cognitive load perceived by CI users during the task (as expressed by their subjective scores) was consistent with and mirrored by their behavioral performance (listening efficiency).

Although listening was clearly more effortful for CI users than for NH participants, no differences in fatigue were found between groups. Similar scores were reported by participants in the FAS questionnaire and the MFQ, suggesting that participants overall experienced low levels of fatigue both in daily life and during task performance. These results support the fact that the experience of listening effort does not necessarily imply the presence of listening-related fatigue. Although it is reasonable to think that there must be a connection between both concepts (Hornsby et al., 2016), there is very little empirical support for a cause-and-effect relationship (McGarrigle et al., 2014; Pichora-Fuller et al., 2016). Indeed, Alhanbali et al.’s (2017) study found low correlations between FAS and EAS scores and concluded that fatigue cannot be reliably predicted from self-reported effort.

Another common assumption is that fatigue could lead to task disengagement (Boksem and Tops, 2008; Hockey, 2013). In the present study our subjects reported low levels of fatigue, and thus, it is not surprising that the levels of disengagement were equally low in both groups. Indeed, previous studies have shown that once CI users engaged in communication they tend to persevere despite experiencing effortful listening (Eckert et al., 2016; Herrmann and Johnsrude, 2020; Perea Pérez et al., 2022).

### 4.2. Drift rates from LBA models: a new metric of listening efficiency

The speed of evidence accumulation (drift rates) toward correct answers (incorrect responses subtracted) was proposed as a measure of participants’ listening efficiency. It was hypothesized that this metric would be able to capture differences in listening performance between groups and conditions. The results of the study confirmed this. As mentioned above, the listening efficiency metric was able to show a significant effect of group in all experimental conditions in sentence trials. Moreover, this measure was sensitive to changes in task demands, showing a declining trend as the task difficulty increased. This tendency was evident (as revealed by negative slopes across conditions) not only in the CI group but also in the control group. This reduction in listening efficiency among NH participants may not be too surprising considering their own subjective ratings during the task— they did perceive an increase in listening effort as the SNR worsened. This may demonstrate the sensitivity of the drift rate parameter in capturing the SATO even when none of the behavioral measures alone could reflect such tendency (i.e., as revealed by similar accuracy and RT distributions of NH participants across conditions in Figure 7). In addition, the lack of effects in null trials supports the validity of the listening efficiency metric at reflecting participants’ performance and cognitive processing load during active listening.

Notably, listening efficiency was correlated with subjective measures of listening effort only in the CI group. The potential of an objective measure of listening performance that is consistent with self-perceived ratings of effort is very promising. Listening efficiency is not subject to individual bias and yet is able to reflect to some extend participants’ perception of listening effort. This is an important quality, given that self-reported measures have been considered more sensitive than other methods to evaluate listening effort (particularly due to changes in task demand), and thus more relevant to audiological contexts (Johnson et al., 2015; Francis and Love, 2019; Visentin et al., 2022). Nonetheless, further investigation is needed to examine the sensitivity of the listening efficiency metric and its relationship with self-reported measures of listening effort.

Although the speech recognition task used in the study was relatively simple for compatibility with the physiological measures simultaneously recorded, such simplicity is not required for the application of the proposed LBA analysis. Listening efficiency can be evaluated using any type of speech recognition task, as long as response time and accuracy are recorded. Therefore, the assessment of listening efficiency may also be relevant to clinical applications. This metric could provide a better evaluation of patients’ performance, taking into account both the speech understanding ability and the cognitive load exerted. This approach could be applied to the test batteries currently used in clinics. Indeed, speech in noise (SIN) tests, such as the Quick Speech in Noise Test (QuickSIN), Hearing in Noise Test (HINT), Bamford-Kowal-Bench SIN Test (BKB-SIN), AzBio Sentence Test, and the City University of New York Sentences (CUNY) are commonly used to assess patients’ intelligibility pre- and after implantation (British Society of Audiology [BSA], 2019). By additionally recording the response time, which may require task adaptation, it would be possible to evaluate CI users’ listening efficiency with the same test batteries already used by audiologists. Nonetheless, we should be mindful of the main limitation of this analysis– LBA models could be analytically costly. Certainly, RT decision models require a considerable post-processing analysis that, although common in research, may be less suitable in clinical environments due to time-constraints. Custom software should be developed to perform such analysis in “real time” and to provide an interpretation of the patients’ performance with respect to the wider CI population. Although this poses a challenge, the development of such software is feasible, just as other clinical solutions were provided in the past to evaluate otoacoustic emissions or auditory evoked potentials.

### 4.3. Individual predictors of listening efficiency

The plausible correlation analysis yielded significant associations between CI users’ listening efficiency and their subjective effort and cognitive ratings, respectively. These findings suggest that better cognition and more positive self-reported listening experiences may be related to higher listening efficiency in CI users.

Indeed, although cognitive tests did not show any significant difference between groups, at the individual level, CI users with worse linguistic closure abilities exhibited poorer listening efficiency. Similar associations were already found by previous studies that concluded that the long-term memory process and lexical access abilities tapped by this test were correlated with speech in noise perception (Kramer et al., 2009; Haumann et al., 2012; Besser et al., 2013; Strand et al., 2018). Haumann’s study even considered the test to be a predictor of better postsurgical speech recognition performance in CI recipients. She suggested that the TRT test should be included in the CI candidacy criteria. The association between the TRT scores and listening efficiency, although moderate (plausible *p* = −0.3), is likely to be present in the CI population with 95% certainty, as revealed by the plausible correlation analysis.

Similarly, the scores of hearing questionnaires describing participants’ hearing abilities (SSQ12) and perceived effort (EAS) in daily life were associated with their efficiency during the task. Higher listening efficiency was positively correlated with better hearing abilities and less perceived effort in everyday life. In the same way, CI users’ self-reported measures of momentary effort and intelligibility during the task seemed to be associated with their listening efficiency. Participants were consciously aware of the level of speech understanding achieved, and thus, their subjective ratings accurately reflected their listening efficiency performance (plausible *p* = 0.8). Conversely, listening efficiency was inversely correlated with task perceived effort (plausible *p* = −0.4).

There is conflicting evidence concerning associations between behavioral and subjective measures of listening effort. While some studies, like the present one, have found correlations between participants’ performance and their effort ratings (Koelewijn et al., 2015; White and Langdon, 2021; Stenbäck et al., 2023), most of the research literature failed to find these associations (Anderson Gosselin and Gagné, 2011; Hornsby, 2013; Strand et al., 2018; Alhanbali et al., 2019; Francis and Love, 2019; McGarrigle et al., 2021; Shields et al., 2023). Although differences in the experimental design (different behavioral task and effort questionnaires) may explain in part the disparity in results, the main difference between previous studies and the present one is how correlations were calculated. Most studies only used one behavioral measure (either accuracy or response time) in their correlation analysis. However, the use of integrated behavioral measures is usually preferred when measuring cognitive or executive functions since they capture the SATO that usually gets lost using response time or accuracy separately (Stafford et al., 2020; Bakun Emesh et al., 2021). The listening efficiency metric used here, being an integrated measure that reflects intelligibility and effort, could have tapped into different domains of the listening effort construct, reflecting perhaps a combination of exerted and (self-) assessed effort (Francis and Love, 2019).

Moreover, the Bayesian nature of the plausible correlation analysis could be more appropriate to explore associations between measures since it overcomes the limitations of multiple testing usually associated with *p*-values in the traditional frequentist approach. Although these could explain the associations found, at this point we can only speculate since there is not enough evidence to prove that this combined metric provides a more comprehensive assessment of listening performance and thus favors correlations with other measures. More research is surely needed to explore the sensitivity of the listening efficiency metric and its relationship with other measures of listening effort.

Finally, the experimental design could have also contributed to improve the coherence and consistency across measures. For instance, the simultaneous acquisition of behavioral and task subjective measures is generally preferred to reduce within-subject variability across measures. Likewise, the ecologically relevant stimuli used in the study could also have favored correlations with subjective measures, given that realistic stimuli are likely to evoke similar perceptions and reactions to those experienced in daily life (and reflected by self-reported questionnaires).

### 4.4. Limitations

It is known that effort measured in the laboratory is likely to differ from the effort experienced in the real world, particularly due to task differences (Pichora-Fuller et al., 2016). Although the main behavioral task was designed to achieve some degree of ecological validity (Keidser et al., 2020) (by using meaningful sentences masked by realistic background noise at most frequent SNR levels), we could not replicate other aspects that play an important role when listening under naturalistic conditions. For instance, the presentation of the sound stimuli did not consider the spatial distribution of sound sources. Both the target speech and the background noise were played using a loudspeaker located in front of the participants. The use of different loudspeakers to present the target and the background noise, as well as a more appropriate distribution of them around participants could have produced a better immersive sound experience of a cafeteria environment. Moreover, no visual cues were available during the listening task. Many CI users rely on visual cues such as facial expressions and lip reading to enhance their speech understanding performance. Thus, the lack of visual cues could have affected participants’ listening efficiency and the effort experienced compared to real life. Even so, this difference may have not been that substantial considering the correlations found between participants’ self-reported measures in daily life and their listening efficiency scores during the task.

The SNRs at which participants performed the main listening task, although representative of everyday life sound scenarios for people with HL (Smeds et al., 2015; Wu et al., 2018), are not likely to pose any listening challenge for NH participants. This low level of task difficulty (positive SNR for all experimental conditions) could have contributed partially to the high listening efficiency exhibited by the NH group, and therefore the great disparity in results with respect to the patient group. The same reasoning could be used to explain partly the lack of correlations found between measures of listening effort within the NH group. One could assume that people with NH may expose themselves to more challenging listening environments in everyday life than the ones reproduced in the behavioral task.

Finally, while the study aimed to detect differences in listening efficiency between NH and CI listeners, it did not account for differences in performance within the CI group (unilateral, bimodal, and bilateral subgroups). Considering the potential benefits of bimodal and bilateral stimulation in speech perception, this generalization of CI recipients may have resulted in wider listening efficiency distributions. Future research could provide further insights into the listening efficiency of CI users as a function of hearing device configuration.

## 5. Conclusion

A LBA model was used to perform a joint analysis of behavioral measures and assess listening efficiency in a group of CI and NH participants. The listening efficiency metric proposed here holds potential as a new outcome measure able to characterize the speed-accuracy trade-off (SATO) of participants’ performance under challenging listening conditions. This metric was sensitive to changes in task demands and able to determine significant differences between groups. Under moderate ecologically relevant levels of background noise, CI users exhibited significantly inferior listening efficiency than their NH peers did in all experimental conditions. Only in the patient group, listening efficiency showed moderate-to-strong correlations with cognitive and self-reported measures of listening effort. This metric should warrant further consideration given its ability to assess both intelligibility and effort. Nonetheless, more research is needed to explore the sensitivity and practical utility of the listening efficiency metric across diverse listening situations.

## Data availability statement

The datasets presented in this article are not readily available because in accordance with our ethical approval, the access to the anonymized study data is limited to the immediate study team and any relevant regulatory authorities. Nonetheless, the anonymized data necessary for reproducing the results of this article can be requested from the corresponding author.

## Ethics statement

The studies involving human participants were reviewed and approved by the University of Nottingham Research Ethics Committee (reference: 247-1902). The patients/participants provided their written informed consent to participate in this study.

## Author contributions

FP, DH, PK, and IW were involved in the conception and design of the study. GN provided mentoring. AZ adapted and provided the Text Reception Threshold used in the study. FP recruited participants, conducted the experiment, collected the data, and wrote the manuscript. FP and IW analyzed the data. DH, PK, GN, AZ, and IW commented on and reviewed the manuscript. All authors contributed to the article and approved the submitted version.

## Footnotes

1
https://rnid.org.uk/

2
https://www.nciua.org.uk/
